# Lysine succinylation as a metabolic switch in cardiovascular diseases: Mechanistic insights and therapeutic perspectives

**DOI:** 10.1016/j.redox.2025.103932

**Published:** 2025-11-14

**Authors:** Fei Mu, Haiyue Zhang, Rui Gong, Rui Lin, Meina Zhao, Xingru Tao, Lei Shang, Miaomiao Xi, Jinyi Zhao, Jingwen Wang

**Affiliations:** a Department of Pharmacy, Xijing Hospital, Fourth Military Medical University, Xi'an, 710032, China; b Department of Health Statistics, Ministry of Education Key Lab of Hazard Assessment and Control in Special Operational Environment, School of Public Health, Fourth Military Medical University, Xi'an, 710032, China; c TANK Medicinal Biology Institute of Xi'an, Xi'an, 710032, China

**Keywords:** Lysine succinylation, Redox homeostasis, Cardiovascular diseases, SIRT5, Cardioprotection

## Abstract

Cardiovascular diseases (CVDs) are life-threatening disorders arising from interactions between genetic and environmental factors, imposing a heavy global health burden with high morbidity and mortality. Emerging evidence suggests that dysregulated epigenetic modifications, particularly lysine succinylation, play a critical role in the pathogenesis of CVD. Characterized by the covalent addition of a succinyl group to lysine residues, succinylation dynamically alters the functions of proteins, including those involved in transcriptional regulation, and directly affects energy metabolism, oxidative stress, inflammation, apoptosis, and fibrosis. This modification has been linked to the development of various CVDs, such as atrial fibrillation, myocardial ischemia-reperfusion injury, myocardial infarction, heart failure, aortic aneurysm and dissection, diabetic cardiomyopathy, hypertrophic cardiomyopathy, and atherosclerosis. Its effects on key biological processes contribute to these conditions through multiple mechanisms. This review systematically summarizes current research on the role of succinylation in cardiovascular pathophysiology, with a particular focus on its function as a “metabolic switch” in CVDs. It further highlights the critical role of succinylation in regulating redox homeostasis and maintaining the balance of SIRT5-mediated desuccinylation. By integrating mechanistic insights from preclinical and clinical studies, we aim to provide a comprehensive framework for understanding the multifaceted roles of succinylation in CVDs and to identify potential therapeutic targets for future translational research.

## Highlights

• Lysine succinylation acts as a key “metabolic switch” in CVDs.
• Redox homeostasis-centered network exhibits dual (pathogenic/protective) pattern in cardiac pathophysiology.
• SIRT5-mediated desuccinylation uniquely sustains in CVDs.
• Mechanistic insights link succinylation to multiple cardiac disorders.
• Targeting succinylation offers promising therapeutic strategies for CVDs.

## 1 Introduction

Cardiovascular diseases (CVDs) remain the primary cause of death worldwide, and their pathogenesis is rooted in intricate molecular networks involving metabolic dysregulation, inflammatory responses, and cellular apoptosis [1]. In recent decades, post-translational modifications (PTMs) have emerged as critical regulators of cellular homeostasis in disease contexts. Among these, lysine succinylation (Ksucc), a dynamic and reversible metabolism-associated modification, is increasingly recognized for its profound involvement in fundamental biological processes, particularly energy metabolism and mitochondrial function. [2,3]. Emerging evidence suggests that Ksucc influences cellular metabolic homeostasis by modulating substrate protein subcellular localization, enzymatic activity, and downstream signaling cascades, with notable roles in cardiovascular system components, including cardiomyocytes, vascular smooth muscle cells, endothelial cells and other related cell types [4,5].

Researchers have measured the concentrations of acyl-coenzyme A (acyl-CoA) compounds in various organs of mice, including the liver, heart, kidney, brain, and muscle. Notably, the findings revealed that different tissues exhibited unique acyl-CoA profiles, with succinyl-coenzyme A (succinyl-CoA) being the predominant acyl-CoA species in the heart [6,7]. Since then, a growing body of research has established a link between Ksucc and CVDs. As a metabolism-sensitive PTM, Ksucc dynamically regulates protein function through the covalent conjugation of succinyl-CoA to the ε-amino group of lysine residues. Notably, in the highly energy-demanding heart, dysregulation of succinylation homeostasis is closely associated with atherosclerosis (AS), myocardial ischemia–reperfusion injury (MI/RI), heart failure (HF), diabetic cardiomyopathy (DbCM), and other cardiac pathologies. This review systematically synthesizes current knowledge on succinylation-mediated metabolic regulatory networks, molecular mechanisms, cross-modification crosstalk, and targeted therapeutic strategies in CVDs, while also outlining future research directions.

## 2 Basic concepts and regulatory mechanisms of lysine succinylation

### 2.1 Discovery, definition and early significance of lysine succinylation

Ksucc is an essential PTM characterized by the covalent attachment of a succinyl group to the ε-amino group of lysine residues, occurring via enzymatic or non-enzymatic mechanisms [8]. This modification was first identified in 2011 by Zhao et al. at the University of Chicago and marked a pivotal breakthrough in the field of PTMs. Using mass spectrometry (MS) combined with protein sequence alignment, they identified succinylated lysine residues in isocitrate dehydrogenase, a key enzyme involved in energy metabolism. To rigorously validate this finding, they employed four independent approaches: Western blot analysis, in vivo labeling with isotopic succinate, MS/MS, and HPLC co-elution of synthetic succinyl-lysine peptide counterparts. The consistency of results across these methods confirmed the existence of Ksucc and demonstrated, for the first time, that evolutionarily conserved lysine residues can undergo succinylation [8].

Subsequent studies in 2012 demonstrated the widespread presence of succinylation in eukaryotic cells, where this modification significantly altered their structure and function [9]. Concurrently, Park et al. [10] reported that metabolic enzymes, including carbamoyl phosphate synthetase 1 and hydroxyacyl-CoA dehydrogenase, exhibit notable succinylation levels in mouse cells. Importantly, they showed that succinylation directly modulates enzymatic activity, establishing succinylation as a conserved regulator of mitochondrial metabolism across the evolutionary spectrum from bacteria to mammals. These early findings shifted the research focus from merely identifying succinylated proteins to exploring their functional impacts. Complementary in vitro studies further support the biological significance of succinylation, which alters the conformational structure of egg white proteins by increasing electrostatic repulsion and reducing surface hydrophobicity, thereby enhancing their thermal stability [11,12]. Given the prevalence of succinylation and the subsequent biochemical alterations, this post-translational modification has garnered increasing interest from the research community.

### 2.2 Chemical characteristics of lysine succinylation

**Fig. 1:**
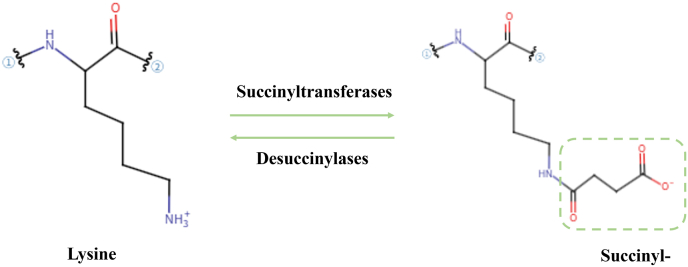
Charge and chemical group alterations in lysine succinylation.

Ksucc is characterized by the covalent conjugation of a succinyl moiety (-CO-CH_2_-CH_2_-COO^-^) to the ε-amino group of lysine residues, resulting in a mass shift of 100.02 Da and altering the charge from +1 to −1 at physiological pH 7.4 (Fig. 1). This distinctive chemical transformation induces profound conformational changes in proteins, thereby limiting their enzymatic activity, subcellular localization, and protein–protein interaction networks. Leveraging high-resolution liquid chromatography–tandem mass spectrometry (LC–MS/MS) and pan-succinyl-lysine antibodies, more than 10,000 succinylation sites have been annotated in the human proteome. Similar to phosphorylation and acetylation, succinylation is a dynamically reversible modification governed by the balance between succinyltransferases and desuccinylases. Table 1 summarizes the key differences between succinylation and other major PTMs. For each PTM type (lysine succinylation, lysine acetylation, lysine methylation, phosphorylation, and ubiquitination), the associated chemical group, core donor, change in molecular weight, and alteration in charge, highlighting its unique role as a metabolic state sensor through direct dependency on succinyl-CoA availability.

**Table 1:** Summarizes key distinctions between succinylation and other major PTMs.

Modification type	Chemical group	Core donor molecule	Molecular weight change (Da)	Charge change
**Lysine succinylation**	-CO-CH_2_-CH_2_-COO^-^	Succinyl-CoA	+100.02	Positive→Negative
**Lysine acetylation**	-CO-CH_3_	Acetyl-CoA	+42.01	Positive→Neutral
**Lysine methylation**	-CH_3_	S-adenosylmethionine	+14.02	No charge change
**Phosphorylation**	-PO_4_	Adenosine triphosphate	+79.97	Increase in negative charge
**Ubiquitination**	Gly76	Ubiquitin	+8564.80	Diverse charge changes

### 2.3 Biological process of succinylation modification

**Fig. 2:**
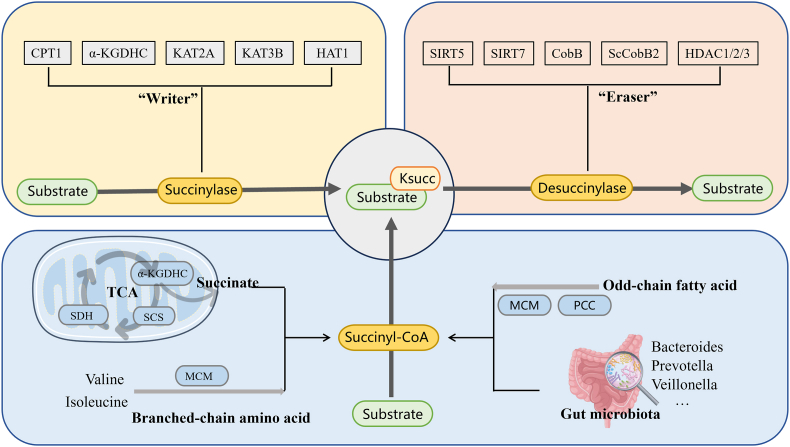
Regulation of succinylation. The mechanisms of succinylation can be divided into two categories: enzymatic modulation and non-enzymatic modulation. *CPT1A* carnitine palmitoyltransferase 1A, *α-KGDHC* α-ketoglutarate dehydrogenase complex, *KAT2A* lysine acetyltransferase 2A, *KAT3B* lysine acetyltransferase 3B, *HAT1* histone acetyltransferase 1, *SIRT5* silent information regulator toxin 5, *SIRT7* silent information regulator toxin 7, *CobB* a sirtuin2-like bacterial lysine deacetylase, *ScCobB2 Streptomyces coelicolor* NAD-dependent protein deacetylase 2, *HDAC1/2/3* histone deacetylase 1/2/3, *Ksucc* lysine succinylation, *TCA* tricarboxylic acid, *SCS* succinyl-CoA synthetase, *SDH* succinate dehydrogenase, *MCM* methylmalonyl-CoA mutase, *PCC* propionyl-CoA carboxylase.

Functioning as a metabolic switch, succinylation dynamically responds to cellular metabolic states, modulating key biological processes. As depicted in Fig. 2, the balance of succinylation is regulated by both enzymatic and non-enzymatic pathways, which act across multiple biological compartments.

#### 2.3.1 Enzymatic lysine succinylation

With advances in research, multiple enzymes with succinyltransferase and desuccinylase activities have been identified. The dynamic balance of succinylation is governed by “Writers” (e.g., CPT1A, α-KGDHC, KAT2A, KAT3B, HAT1) and “Erasers” (e.g., SIRT5, SIRT7, CobB, ScCobB2, HDAC1/2/3) [2,9,[13], [14], [15]]. Specifically, carnitine palmitoyltransferase 1A (CPT1A), a lysine succinyltransferase, inhibits enolase 1 activity and promotes cell proliferation under glutamine depletion, independent of its classical carnitine palmitoyltransferase activity [16]. The α-ketoglutarate dehydrogenase complex (α-KGDHC) possesses *trans*-succinyltransferase activity, which mediates succinylation in an α-ketoglutarate-dependent manner, thereby regulating cellular metabolic processes [17]. Nuclear α-ketoglutarate dehydrogenase-coupled lysine acetyltransferase 2A (KAT2A) and lysine acetyltransferase 3B (KAT3B) can succinylate histone H3 on lysine 79 (H3K79), and this modification occurs particularly frequently near gene transcription start sites [13]. Histone acetyltransferase 1 (HAT1) catalyzes succinylation at H3K122, contributing to epigenetic regulation and gene expression, and also catalyzes succinylation at K99 of the non-histone protein phosphoglyceromutase 1, thereby supporting glycolysis [18]. SIRT5 and SIRT7 are the most commonly identified desuccinylases in eukaryotes [19,20]. CobB, CobB, a sirtuin 2–like bacterial lysine deacetylase, was the first prokaryotic desuccinylase identified to have both deacetylase and desuccinylase activities [21]. Similarly, another sirtuin-like protein, ScCobB2, has been confirmed as a specific desuccinylase in *Streptomyces coelicolor* [22]. Class I HDACs (HDAC1/2/3) are the major histone desuccinylases in mammalian cells, and their purified endogenous forms exhibit vigorous histone desuccinylase activity in vitro [23].

#### 2.3.2 Non-enzymatic lysine succinylation by succinyl-CoA

Ksucc can occur through a non-enzymatic process, in which succinyl-CoA serves as the primary donor of succinyl groups. Succinyl-CoA is found in multiple tissues, with the highest abundance in the heart. The common production pathways are as follows [6,24]. In the mitochondrial endogenous pathway, the tricarboxylic acid (TCA) cycle plays a critical role, with the α-ketoglutarate dehydrogenase complex (α-KGDHC), succinate dehydrogenase (SDH), and succinyl-CoA synthetase (SCS) acting as key catalysts [10,13,25]. Specifically, α-KGDHC catalyzes the conversion of α-ketoglutarate to succinyl-CoA, not only providing substrates for subsequent TCA cycle reactions but also supplying an essential donor for succinylation. Notably, both the TCA cycle and electron transport chain rely on SDH, and the desuccinylation of SDH modulates its enzymatic activity, further affecting mitochondrial respiration. Branched-chain amino acid metabolism is another vital route for mitochondrial succinyl-CoA synthesis. A study by Takada et al. revealed that isoleucine and valine can generate succinyl-CoA via the methylmalonyl-CoA mutase (MCM) pathway, further expanding the sources of mitochondrial succinyl-CoA [26]. In the mitochondrial exogenous pathway, odd-chain fatty acids are metabolized so that propionyl-CoA, generated during β-oxidation, is converted to methylmalonyl-CoA by propionyl-CoA carboxylase (PCC). It is then isomerized to succinyl-CoA by MCM. Gut microbiota metabolism also merits attention, as specific microbial taxa (e.g., *Bacteroides*, *Prevotella*, *Veillonella*) ferment dietary fiber to produce succinate, which enters cardiomyocytes through the portal vein after absorption, providing substrates for intracellular succinyl-CoA synthesis and highlighting the potential connection between gut microbiota metabolism and succinylation in cardiomyocytes [27,28].

### 2.4 Succinylation modification of histone proteins and non-histone proteins

Proteins modified by succinylation can be classified into histones and non-histones. Histone succinylation primarily regulates epigenetic processes by influencing gene expression through chromatin structure modification, whereas non-histone succinylation extensively regulates cellular processes, particularly involving key enzymes in central metabolic pathways [9,29].

In the regulatory mechanism of histone succinylation, Wong et al. were the first to reveal that HDAC1/2/3, rather than the sirtuin family, act as the major histone desuccinylases, extensively catalyzing desuccinylation at sites such as histones H3K14 and H3K23. The minimal core complexes of HDAC1 and HDAC3 are sufficient to exert histone desuccinylase activity, providing new insights into the role of histone succinylation in transcriptional regulation [23]. SIRT7, primarily localized in the nucleus, is also considered to be involved in histone desuccinylation. Studies have shown that SIRT7-catalyzed H3K122 desuccinylation plays a critical role in the DNA damage response and cell survival [19,30].

**Fig. 3:**
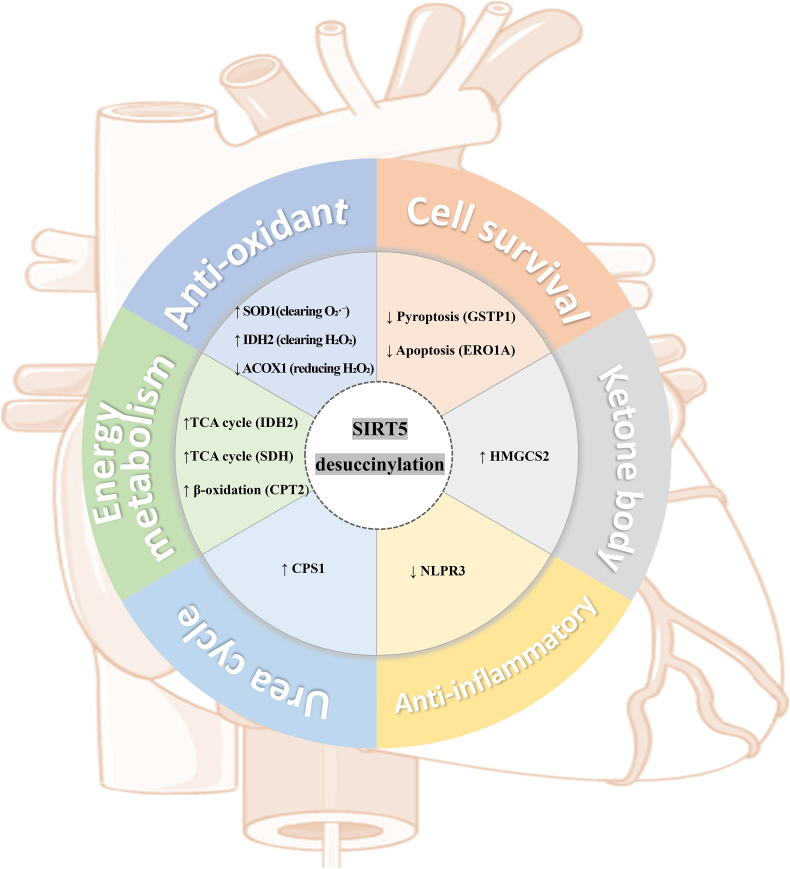
Core mechanisms of Sirt5-mediated desuccinylation in cardioprotection. *IDH2* isocitrate dehydrogenase 2, *SDH* succinate dehydrogenase complex, *CPT2* carnitine palmitoyltransferase 2, *SOD1* superoxide dismutase 1, *IDH2* isocitrate dehydrogenase 2, *ACOX1* acyl-CoA oxidase 1, *GSTP1* glutathione S-transferase Pi 1, *ERO1A* endoplasmic reticulum oxidoreductase 1 alpha, *HMGCS2* 3-hydroxy-3-methylglutaryl-CoA synthase 2, *NLRP3* NOD-like receptor family pyrin domain containing 3, *CPS1* carbamoyl-phosphate synthetase 1.

In the regulatory mechanism of histone succinylation, notably, SIRT5, a core desuccinylase belonging to the sirtuin protein family, exerts its activity in an NAD^+^-dependent manner. Highly expressed in the heart, liver, and kidney, SIRT5 regulates multiple metabolic enzymes by removing succinyl groups [31]. The known regulatory mechanisms and target sites are shown in Fig. 3. **Energy metabolism regulation**: SIRT5 activates isocitrate dehydrogenase 2 (IDH2) and SDH through desuccinylation to maintain TCA cycle flux. It also desuccinylates carnitine palmitoyltransferase 2 (CPT2) to promote fatty acid β-oxidation, thereby alleviating lipotoxicity in the diabetic myocardium [32]. **Anti-oxidant defense:** SIRT5 desuccinylates superoxide dismutase 1 (SOD1) to enhance its ROS-scavenging capacity, specifically by clearing the superoxide anion (O_2_**·**^-^) [33], activates IDH2 via desuccinylation to promote nicotinamide adenine dinucleotide phosphate (NADPH) and glutathione production, thereby improving hydrogen peroxide (H_2_O_2_) clearance and oxidative stress resistance [34], and inhibits acyl-CoA oxidase 1 (ACOX1) through desuccinylation, reducing intracellular H_2_O_2_ generation [35]. **Cell survival protection:** SIRT5 inhibits pyroptosis (through GSTP1) and apoptosis (through ERO1A) to preserve myocardial cell viability [36]. **Ketone body:** SIRT5 modulates succinylation of 3-hydroxy-3-methylglutaryl-CoA synthase 2 (HMGCS2), a key rate-limiting enzyme involved in ketogenesis, both in vivo and in vitro. SIRT5-mediated desuccinylation activates HMGCS2 to promote ketone body production, providing an alternative energy source for the myocardium [37]. **Anti-inflammatory function:** Through desuccinylation, SIRT5 inhibits the assembly and activation of the NOD-like receptor protein 3 (NLRP3) inflammasome, thereby reducing myocardial inflammation induced by high glucose or lipotoxicity. **Urea cycle:** The deletion of SIRT5 in mice appears to increase succinylation of carbamoyl phosphate synthase 1(CPS1), a known target of SIRT5 [38].

### 2.5 Regulatory mechanisms of lysine succinylation in cardiovascular diseases

#### 2.5.1 Regulation of energy metabolism

The functional integrity of mitochondria is vital for cardiac homeostasis [39,40]. Research has shown that mitochondria-associated proteins are frequently subjected to succinylation modifications, which directly affect energy metabolic efficiency by regulating key metabolic pathways [41]. For example, changes in the succinylation levels of multiple enzymes in the TCA cycle can significantly suppress the cycle's flux, leading to energy supply disorders in cardiomyocytes, and thereby contributing to the development of CVDs. Specifically, succinylation disrupts the balance between ATP production and substrate utilization by modifying the enzymes involved in the TCA cycle, fatty acid oxidation (FAO), and ketone body metabolism [42,43]. **Isocitrate Dehydrogenase 2 (IDH2)**, a key enzyme involved in mitochondrial energy metabolism, is significantly influenced by succinylation status. Promoting its desuccinylation restores the conversion efficiency of isocitrate to α-ketoglutarate, significantly enhancing TCA cycle flux and ATP synthesis capacity, thereby alleviating myocardial energy metabolic disorders [25]. Chang et al. [44] demonstrated that quercetin may mediate IDH2 desuccinylation by activating SIRT5, maintaining mitochondrial homeostasis, reducing myocardial fibrosis, and lowering the risk of HF in inflammatory models. **Succinate Dehydrogenase A (SDHA)** is a core subunit of the respiratory chain complex II. Succinylation of SDHA inhibits the conversion of succinate to fumarate, blocking electron transport and reducing ATP production. Restoring the desuccinylated state of SDHA safeguards the respiratory chain function. It maintains the myocardial energy supply, thus playing a critical role in preventing and treating CVDs associated with mitochondrial dysfunction [45]. **Pyruvate Dehydrogenase Complex (PDC)** catalyzes the oxidation of pyruvate to acetyl-CoA (the substrate for TCA cycle entry). Its activity is inhibited by desuccinylases, indicating that succinylation may regulate TCA cycle entry flux by modulating PDC activity [10]. **Carnitine Palmitoyl transferase 2 (CPT2)** mediates the transport of long-chain acylcarnitines into the mitochondrial matrix to initiate β-oxidation and supply energy to cardiomyocytes. Studies have demonstrated that CPT2 succinylation significantly impairs its transport activity, and CPT2 exhibits reduced affinity for its substrate (long-chain acylcarnitines), thereby impairing mitochondrial fatty acid uptake for oxidation. Collectively, these effects result in diminished myocardial energy supply, fatty acid accumulation, and lipotoxic damage, ultimately compromising cardiomyocyte function. The level of CPT2 succinylation is closely correlated with the progression of CVDs, such as myocardial ischemia and hypertrophic cardiomyopathy. Reducing its succinylation level is expected to restore its transport function, enhance β-oxidation, improve myocardial energy metabolism, and provide new therapeutic targets for the treatment of CVDs [32].

#### 2.5.2 Regulation of inflammation

Uncontrolled inflammatory responses are critical pathological processes in CVDs. Succinylation modulates inflammation-related signaling pathways, and the succinylation of specific inflammatory factors has a significant influence on gene expression and protein release. Aberrant succinylation of proteins involved in inflammatory pathways can either hyperactivate or fail to terminate inflammation, thereby driving the progression of CVD. The NLRP3 inflammasome plays a pivotal role in inflammation, and its succinylation status regulates inflammasome assembly and activation. Studies have shown that succinylation of NLRP3 promotes its binding to the adapter protein ASC, thereby activating caspase-1 and inducing the maturation and release of pro-inflammatory cytokines, such as interleukin-1β (IL-1β), which exacerbates inflammation. The promotion of NLRP3 desuccinylation inhibits inflammasome activation, reduces cytokine release, and alleviates inflammatory damage in CVDs, offering novel anti-inflammatory therapeutic strategies. In addition, Zhuo et al. [46] demonstrated that CD44 in macrophages serves as an inflammation-associated receptor for TcdB/FBD. SUCLG2 inhibition promotes CD44 K158 succinylation, enhances NF-κB translocation and transcriptional activity, and triggers inflammatory responses.

#### 2.5.3 Regulation of oxidative stress

**Fig. 4:**
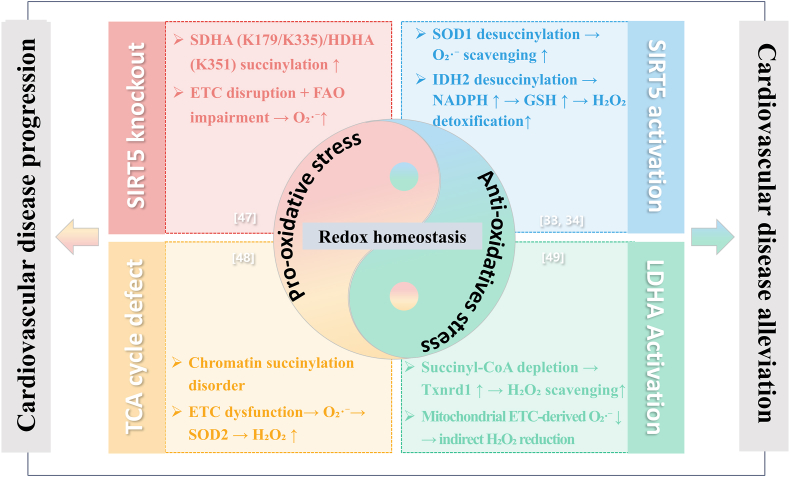
Succinylation regulates intracellular redox status, dictating cardiovascular disease progression or alleviation via a bipolar mechanism centered on redox homeostasis. *SIRT5* silent information regulator toxin 5, *SDHA* succinate dehydrogenase A, *HDHA* hydroxyacyl-CoA dehydrogenase subunit A, *ETC* electron transport chain, *FAO* fatty acid oxidation, *O*_*2*_·- superoxide anion, *TCA* tricarboxylic acid, *SOD2* superoxide dismutase 2, *H*_*2*_*O*_*2*_ hydrogen peroxide, *SOD1* superoxide dismutase 1, *NADPH* nicotinamide adenine dinucleotide phosphate, *GSH* glutathione, *LDHA* lactate dehydrogenase A, *Txnrd1* thioredoxin reductase 1.

Succinylation interacts with oxidative stress–related proteins to regulate intracellular redox status, thereby influencing the progression or mitigation of CVDs. This regulatory network centers on redox homeostasis and exhibits a dual pattern: mechanisms that disrupt redox balance promote disease progression, whereas those that preserve redox balance alleviate cardiovascular pathology (Fig. 4).

Two key pathways contribute to redox imbalance and oxidative stress, accelerating CVD progression. Aberrant succinylation of oxidative metabolic proteins represents a major alteration following SIRT5 knockout, with validated cardiac targets including lysine 179 and 335 of SDHA and lysine 351 of hydroxyacyl-CoA dehydrogenase subunit A (HDHA). Functional impairment of these enzymes disrupts the ETC and hinders complete FAO, thereby inducing oxidative stress [47]. Additionally, Smestad J et al. reported that TCA cycle defects alter chromatin succinylation patterns; specifically, loss of SDH leads to mitochondrial ETC dysfunction and excessive generation of O_2_**·**^-^, which are subsequently converted to H_2_O_2_ by mitochondrial superoxide dismutase 2 (SOD2). These ROS mediate oxidative stress, resulting in DNA repair deficiencies and heightened sensitivity to genotoxic agents [48]. In contrast, two major pathways preserve redox homeostasis via targeted succinylation and desuccinylation, thereby mitigating cardiovascular injury. Lin et al. demonstrated that SOD1 is subject to succinylation, which reduces its enzymatic activity, whereas SIRT5 binds to SOD1, mediating its desuccinylation and reactivation. As SOD1 catalyzes the conversion of O_2_·^-^ into oxygen and H_2_O_2_, co-expression of SIRT5 with SOD1 markedly enhances the clearance of O_2_**·**^-^. Experimental evidence shows that SIRT5 and SOD1 co-overexpression reduces intracellular O_2_·^-^ levels by 43 %, compared with a 19 % reduction observed with SOD1 overexpression alone [33]. Furthermore, SIRT5 desuccinylates IDH2 at K413, enhancing its activity and promoting NADPH generation. This elevation in NADPH increases levels of reduced glutathione (GSH) and facilitates H_2_O_2_ detoxification, thereby protecting cardiomyocytes from oxidative injury [34]. Within this oxidative stress regulatory network, metabolomic, proteomic, and co-IP analyses collectively revealed that LDHA-mediated depletion of succinyl-CoA inhibits succinylation-dependent ubiquitination of thioredoxin reductase 1 (Txnrd1). This cascade mitigates the overproduction of two major ROS subtypes: (i) H_2_O_2_, as Txnrd1 maintains reduced Trx to assist Prxs in H_2_O_2_ decomposition—confirmed by DCFH-DA probing, which showed that LDHA/Txnrd1 overexpression lowers H_2_O_2_ levels, while Txnrd1 inhibition reverses this effect—and (ii) O_2_·^-^, as LDHA reduces mitochondrial ETC-derived O_2_·^-^, which SOD2 dismutates to H_2_O_2_, indirectly decreasing total H_2_O_2_ accumulation. This coordinated activity alleviates oxidative damage, releases cell cycle arrest, and promotes cardiomyocyte proliferation [49]. Together, these pro-oxidative and antioxidative succinylation regulatory processes modulate redox homeostasis, thereby influencing the progression or remission of CVDs. Consequently, succinylation represents a pivotal regulatory node for redox balance and a promising therapeutic target for CVD intervention, warranting further in-depth investigation.

#### 2.5.4 Regulation of apoptosis and fibrosis

Metabolic alterations, such as succinate accumulation, serve as key direct factors that trigger the activation of cardiac fibroblasts and promote their resistance to apoptosis. Mechanistic investigations confirmed that SIRT5 knockdown could trigger the apoptotic program in endothelial cells (HUVECs) by facilitating the succinylation modification of endoplasmic reticulum oxidoreductase 1α (ERO1A) at Lys396 [36]. Additionally, the age-related buildup of succinate encourages cardiac fibrogenesis through a specific mechanistic process. In this process, succinate binds to its receptor (SUCNR1/GPR91) on the membranes of fibroblasts, which activates the downstream p38-MAPK signaling pathway. This activation leads to the succinylation of tetrameric pyruvate kinase M2 (PKM2), causing it to dissociate into dimers. These dimers then move to both the nucleus and the mitochondria. In the nucleus, succinylated PKM2 dimers form a transcription complex with hypoxia-inducible factor-1α (HIF-1α), enhancing DNA binding of HIF-1α and upregulating fibrogenic genes (e.g., collagen I/III) to promote extracellular matrix synthesis. Simultaneously, the mitochondrial translocation of succinylated PKM2 dimers enhances their binding to voltage-dependent anion channel 1 (VDAC1), leading to ubiquitin-mediated degradation via the SYVN1 E3 ligase. This process decreases cytochrome *c* release from the mitochondria, prevents fibroblast apoptosis, and supports fibrogenic activity. Together, these mechanisms establish a feed-forward loop in which succinate-driven PKM2 dimerization coordinates collagen synthesis and cell survival, exacerbating age-related cardiac apoptosis and fibrosis [50,51].

## 3 Functional roles of lysine succinylation in cardiovascular diseases

**Fig. 5:**
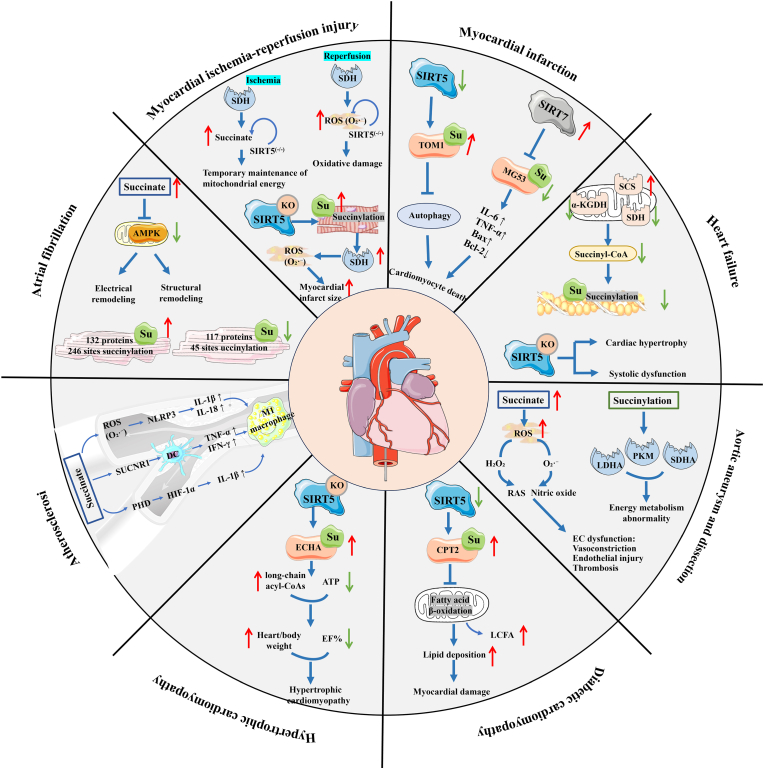
Panoramic map of the succinylation metabolic network and its association with cardiovascular diseases. *Su* succinylation, *AMPK* adenosine 5′-monophosphate -activated protein kinase, *SDH* succinate dehydrogenase, *SIRT5* silent information regulator toxin 5, *SIRT7* silent information regulator toxin 7, *TOM1* tumour susceptibility gene 1, *MG53* mitsugumin 53, *α-KGDH* α-ketoglutarate dehydrogenase, *SCS* succinyl-CoA synthetase, *ROS* reactive oxygen species, *RAS* renin-angiotensin system, *LDHA* lactate dehydrogenase A, *PKM* pyruvate kinase M, *SDHA* succinate dehydrogenase complex subunit A, *CPT2* carnitine palmitoyltransferase 2, *LCFA* long-chain fatty acid, *ECHA* enoyl-CoA hydratase A, *ATP* adenosine triphosphate, *EF* ejection fraction, *NLRP3* NOD-like receptor family pyrin domain containing 3, *SUCNR1* succinate receptor 1, *DC* dendritic cell, *PHD* prolyl hydroxylase domain, *HIF-α* hypoxia-inducible factor alpha.

As illustrated in Fig. 5, Ksucc acts as a metabolic switch and exerts pleiotropic regulatory effects that maintain balance in a range of CVDs, including atrial fibrillation (AF), myocardial ischemia-reperfusion injury (MI/RI), myocardial infarction (MI), heart failure (HF), aortic aneurysm/dissection (AAD), diabetic cardiomyopathy (DbCM), hypertrophic cardiomyopathy (HCM), and atherosclerosis (AS).

### 3.1 Atrial fibrillation

AF is the most common arrhythmia associated with high energy demand in cardiomyocytes and impaired energy production [52]. In 2018, Bai et al. proposed that reversible succinylation occurring in the mitochondria might regulate impaired energy metabolism in AF [53]. Subsequently, in 2019, the same team analyzed the proteomic profiles and succinylation status of left atrial appendage tissue samples from 18 patients undergoing cardiac valve surgery, using dimethyl labeling combined with high-performance liquid chromatography-tandem mass spectrometry and western blotting. Their results revealed that in patients with AF, 246 sites across 132 proteins showed upregulated succinylation, whereas 45 sites in 117 proteins showed downregulated succinylation. These differentially succinylated sites were primarily enriched in energy metabolism-related proteins, suggesting that these proteins participate in the pathogenesis of AF in patients with valvular heart disease. This study provides new insights into the mechanisms and therapeutic targets of AF [54].

In an experimental model of ventricular fibrillation in large white pigs, succinate accumulation was observed in cardiomyocytes [55]. Succinate overload impairs the AMPK signaling pathway and mitochondrial function, thereby inducing atrial electrical and structural remodeling and increasing the risk of AF. Additionally, studies have shown that activation of AMPK with its agonist 5-aminoimidazole-4-carboxamide ribonucleotide (AICAR) effectively reduces succinate-induced AF susceptibility and related pathological changes, indicating that succinate may serve as a novel biomarker and therapeutic target for AF [52].

### 3.2 Myocardial ischemia-reperfusion injury

A 2014 study published in Nature demonstrated that succinate accumulates markedly during ischemia and is rapidly metabolized as mitochondrial O_2_**·**^-^ production increases during reperfusion. This indicates that succinate serves as the primary driver of mitochondrial O_2_**·**^-^ generation during reperfusion and underlies IR in various tissues [56]. During ischemia, the TCA cycle intermediate succinate selectively accumulates due to the reversal of SDH. In the reperfusion phase, accumulated succinate is re-oxidized by SDH, driving reverse electron transport through mitochondrial complex I and generating excessive O_2_**·**^-^, which in turn leads to oxidative damage, cell death, and aberrant immune responses [25]. Liu et al. reported that exogenous NAD ^+^ administration plays a crucial role in metabolism. It enhanced SIRT5-mediated SDHα desuccinylation and the GSH*/*GSSG ratio, while reducing SDHA activity, thereby mitigating succinate accumulation during ischemia and decreasing succinate consumption during reperfusion. This process ultimately reduces the production of ROS, primarily O_2_·^-^, which helps provide cardioprotective effects [57].

Furthermore, studies have revealed that during ischemia, succinate accumulation through mitochondrial complex II may sustain a partial energy supply through substrate-level phosphorylation. Conversely, during reperfusion, the oxidation of accumulated succinate through mitochondrial complex II drives the O_2_·^-^ burst and subsequent cellular damage. Therefore, the inhibition of mitochondrial complex II activity during the early reperfusion phase can effectively attenuate such injury [58,59]. Boylston et al. [60] demonstrated in in vitro Langendorff studies of SIRT5-knockout mice after I/R that succinylated proteins accumulate in cardiac metabolic pathways. Specifically, 184 such proteins were enriched in core pathways, including oxidative phosphorylation, fatty acid β-oxidation, and the TCA cycle. Moreover, these proteins exhibited higher succinylation levels in knockout mice; for example, succinylation at the SDH subunit DHSA's K179/K335 was only detected in Sirt5^(−/−)^mice. These alterations synergistically exacerbate O_2_·^-^ generation via mitochondrial electron transport chain (ETC) complex I reverse electron transport, thereby amplifying myocardial infarction severity. They further showed that pretreatment with dimethyl malonate, a competitive inhibitor of SDH, could restore I/R injury in Sirt5^(−/−)^ hearts to the wild-type (WT) level. This finding indicates that the change in SDH activity is the cause of injury. Ou et al. [61] reported that SIRT5-deficient adipose-derived mesenchymal stem cells (ADMSCs) exhibited increased protein succinylation, diminished mitochondrial respiratory capacity, and perturbed glucose metabolism, which in turn accelerated vascular proliferation. Given the critical role of succinate in MI/RI, targeting the succinate production pathways offers a potential therapeutic option [62]. In vivo experiments demonstrated that administering AICAR and aminooxyacetate reduced ischemic succinate production. Specifically, AICAR inhibits the conversion of α-ketoglutarate to succinate, while aminooxyacetate blocks succinate-mediated electron acceptance by coenzyme Q [56]. Furthermore, the complex II inhibitor dimethyl malonate decreases ischemic succinate levels and O_2_·^-^ production, subsequently reducing infarct size. Infusion of dimethyl malonate into isolated rat hearts also protected against MI/RI [60,63]. These findings highlight the potential of interventions against harmful succinate overproduction; however, further clinical evaluation is warranted.

### 3.3 Myocardial infarction

The relationship between acute myocardial infarction and succinylation is primarily reflected in the regulation of Ksucc during myocardial energy metabolism, inflammation, and tissue repair. Zhou et al. reported that succinylation occurs in mammalian serum proteins. Compared to healthy volunteers, the abundance of protein succinylation, malonylation, and glutarylation in the peripheral serum of patients with ST-segment elevation myocardial infarction (STEMI) was significantly reduced (*p* < 0.05), suggesting that these modifications may serve as novel biomarkers [64]. In a mouse model of acute myocardial infarction, SIRT5 expression was significantly upregulated in the liver. To further explore the mechanism underlying the alleviation of acute cardiac ischemia, liver-specific SIRT5-overexpressing mice were generated and subjected to myocardial ischemia modeling alongside WT mice. Results revealed that, compared to WT controls, liver-specific SIRT5-overexpressing mice exhibited markedly reduced MI area and degree of myocardial fibrosis, accompanied by significantly elevated levels of secreted fibroblast growth factor 21 (FGF21) in the circulation. These findings suggest that SIRT5 exerts cardioprotective effects through a novel liver-cardiac crosstalk mechanism in MI injury [65]. SIRT5 was found to directly interact with the target of myb1 membrane trafficking protein (TOM1) and reduce TOM1 succinylation at the K48 site. Consistently, shSIRT5 treatment decreased total TOM1 levels while increasing the levels of succinylated TOM1 [66]. Upon myocardial injury, the desuccinylase activity of SIRT7 is enhanced, leading to the desuccinylation of MG53 at Lys130. Concomitantly, levels of inflammatory cytokines IL-6 and TNF-α increase, accompanied by upregulation of Bax and downregulation of Bcl-2, while KAT3B-facilitated promotion of MG53 succinylation at K130 sites hinders MG53 ubiquitination and elevates its protein abundance, thereby alleviating cardiomyocyte damage. These observations indicate that MG53 is a potential therapeutic target for MI [67].

### 3.4 Heart failure

The high prevalence of HF continues to impose substantial mortality burdens and unfavorable prognoses in affected individuals [68]. Against this backdrop, succinylation has emerged as a critical regulator of the pathological processes in HF. Quantitative LC-MS/MS proteomic profiling demonstrates a significant reduction in myocardial myofibrillar protein succinylation levels in failing hearts, accompanied by decreased activities of α-ketoglutarate dehydrogenase (α-KGDH) and SDH, but increased SCS activity. This metabolic dysregulation likely enhances succinyl-CoA consumption while reducing its production, consequently leading to the hypo-succinylation of myofibrillar proteins. Notably, patients with ischemic cardiomyopathy exhibit a 65 % decrease in SIRT5 protein expression; however, the observed hyposuccinylation was independent of SIRT5 activity. This implies that non-enzymatic mechanisms, such as altered succinyl-CoA homeostasis, are the dominant regulators. Targeted modulation of succinyl-CoA metabolic flux therefore holds promise as a novel therapeutic strategy for HF [42,69,70].

Impaired succinyl-CoA metabolism disrupts TCA cycle flux and ATP synthesis [26]. Notably, SDH is required for maintaining myocardial homeostasis during FAO/glycolysis. When the subunit b or c of SDH was deleted specifically in cardiomyocytes of mice, they developed dilated cardiomyopathy and HF [71]. In advanced HF, the heart undergoes metabolic reprogramming and preferentially utilizes ketone bodies for energy, with β-hydroxybutyrate alleviating oxidative stress by promoting the expression of the antioxidant enzyme superoxide dismutase [72,73]. Furthermore, mice with SIRT5 deficiency develop cardiac hypertrophy and contractile dysfunction, indicating that excessive succinylation promotes HF progression by impairing mitochondrial protein function [6,47]. However, since the pathogenesis of HF with preserved ejection fraction (HFpEF) remains unclear, and succinylation plays a central role in myocardial stress regulation. Thus, further studies are needed to clarify the association between succinylation dysregulation and HFpEF progression.

### 3.5 Aortic aneurysm and dissection

AAD, a life-threatening cardiovascular condition, hinders diagnosis and monitoring due to its asymptomatic nature, raising the risk of aortic wall dissection and rupture [74,75]. Studies have indicated that succinate plays a pivotal role in the pathogenesis of AAD. A research team led by Professor Le-Min Zheng from Peking University, using untargeted metabolomics analysis, revealed elevated succinate levels in patients with aortic aneurysms. Notably, this trend persisted when compared with patients with acute myocardial infarction and pulmonary embolism, suggesting that succinate may serve as a biomarker for the diagnosis of aortic aneurysms and help distinguish AAD from acute myocardial infarction and pulmonary embolism in patients presenting with chest pain [76]. Succinate induces the production of macrophage-derived ROS, specifically O_2_·^-^ and its dismutation product H_2_O_2_, promoting the progression of aortic aneurysms and dissection. It further exacerbates endothelial dysfunction by upregulating O_2_·^-^ and H_2_O_2_ levels: O_2_·^-^ impairs nitric oxide-mediated vasodilation, whereas H_2_O_2_ activates the renin-angiotensin system (RAS) and enhances thrombogenesis [28]. Proteomic analysis revealed that succinylation levels were significantly elevated in the aortic tissues of patients with thoracic aortic aneurysms and dissections, involving 197 differentially expressed succinylated proteins. These proteins were primarily enriched in mitochondria and the cytoplasm, and participated in metabolic pathways such as carbon metabolism, amino acid catabolism, and fatty acid β-oxidation [77]. Succinylation may affect energy metabolism in muscle cells by altering the activity of key metabolic enzymes (such as PKM, LDHA, and SDHA), thereby contributing to structural and functional abnormalities of the aortic wall [5,78]. Therefore, the aberrant expression of succinylated proteins may serve as diagnostic biomarkers for aortic diseases, and regulating relevant modifying enzymes could become a therapeutic strategy.

### 3.6 Diabetic cardiomyopathy

DbCM is a severe complication of cardiac injury in patients with diabetes and is characterized by the accumulation of toxic lipid metabolites in the myocardial cells, leading to cardiac dysfunction [41]. Wu et al. demonstrated that SIRT5 expression is significantly reduced in a DbCM mouse model. Succinylation of CPT2 at Lys424 impairs its binding ability to carnitine, preventing long-chain fatty acids from entering mitochondrial β-oxidation. This exacerbates lipid deposition and aggravates myocardial injury [32]. Meanwhile, Wei et al. demonstrated that SIRT5 deficiency exacerbates cardiac injury in DbCM mice and high-glucose-induced myocardial cell damage [79]. Consequently, SIRT5-mediated Ksucc has emerged as a promising therapeutic target for DbCM. Investigating the therapeutic efficacy of SIRT5 activators, such as investin and resveratrol, in DbCM and other lipid-related metabolic disorders has significant clinical implications. It also involves elucidating whether succinylation serves as a mediating mechanism in these pathologies [41].

### 3.7 Hypertrophic cardiomyopathy

Cardiac hypertrophy is associated with abnormal fatty acid metabolism. Studies have shown that in SIRT5-deficient mice under fasting conditions, impaired desuccinylation reduces the activity of enoyl-CoA hydratase α subunit (ECHA), which leads to the accumulation of long-chain acyl-CoA, decreased ATP levels, and ultimately hypertrophic cardiomyopathy [6]. Specifically, using a SIRT5 knockout (SIRT5 KO) mouse model combined with a metabolomics-assisted proteomics approach, researchers found that lysine-succinylated proteins primarily accumulated in the heart, with the highest number of succinylation sites in ECHA (28 lysine residues). This indicates a close relationship between fatty acid β-oxidation and cardiac succinylated proteins, providing new insights into the pathogenesis of cardiac hypertrophy.

Additionally, Hershberger et al. [47] developed a tamoxifen-inducible heart-specific SIRT5 KO mouse model. Through quantitative succinyl proteomic analysis of the heart, they found that succinylation of oxidative metabolic proteins increased 15–31 weeks after SIRT5 ablation. Notably, heart-specific SIRT5 knockout mice showed no significant difference in survival compared to controls when exposed to chronic pressure overload, in contrast to whole-body SIRT5 KO mice. This suggests that the survival of SIRT5 KO mice may be dictated by the multi-tissue or prenatal effects of SIRT5.

### 3.8 Atherosclerosis

Accumulating evidence indicates that succinate levels rise significantly under hyperlipidemic conditions, driven by elevated glucose, lipopolysaccharide, and lipid levels. These metabolic abnormalities impair succinate dehydrogenase activity, thereby causing succinate to accumulate as a substrate in the reaction [28,80,81]. Additionally, succinate stimulates dendritic cells (DC) and M1 macrophages to produce pro-inflammatory cytokines, thereby accelerating AS. Elevated succinate levels increase ROS production (particularly O_2_·^-^), activate succinate receptor 1 (SUCNR1), and upregulate hypoxia-inducible factor-1α (HIF-1α) through inhibiting prolyl hydroxylase domain (PHD), thereby triggering the production of tumor necrosis factor-α (TNF-α), interferon-γ (IFN-γ), and interleukin-1β (IL-1β) [82,83]. Concurrently, succinylation in vascular smooth muscle cells activates the NLRP3 inflammasome, promoting the release of IL-1β and IL-18 [28]. Thus, therapeutic strategies targeting succinate hold promise as a novel paradigm for managing AS. Studies have demonstrated that inhibiting succinate-induced NF-κB activation can attenuate matrix metalloproteinase-9 (MMP-9) activity and suppress vascular smooth muscle cell migration, thereby protecting against plaque rupture [28].

Additionally, cinnamaldehyde modulates NLRP3 inflammasome formation via the succinate/HIF-1α axis to regulate IL-1β production, thereby suppressing inflammatory responses [84]. Furthermore, nanodrug delivery systems, such as mangiferin-loaded N-succinyl chitosan-alginate nanoparticles, can target and inhibit protein succinylation and reduce plaque formation [85]. Therefore, targeting succinylation may be an effective approach for treating AS.

### 3.9 Others

Coronary microembolization refers to embolization that occurs within the coronary microcirculation. Studies have demonstrated that desuccinylation induces the accumulation of TRMT10C in the nucleus, which prior studies have shown exacerbates coronary microembolism progression through its m1A modification function [86].

## 4 Therapeutic exploratory

Although succinylation has gradually emerged as a research hotspot in the field of epigenetics related to CVDs, investigations into relevant therapeutic strategies remain in their infancy.

As shown in Table 2, in the field of therapeutic intervention, SIRT5 agonists and succinylase inhibitors have been shown to reverse myocardial energy metabolic abnormalities and attenuate ROS production in animal models [87,88]. The development of SIRT5-specific agonists can enhance desuccinylation activity, improve mitochondrial function, and mitigate myocardial injury [31]. Administration of exogenous NAD promotes Sirt5-mediated SDH-a desuccinylation by regulating the dynamic balance of succinylation, this desuccinylation process further reduces SDH-a activity, which in turn attenuates the succinate accumulation during ischemia and its depleting rate during reperfusion and finally alleviated O_2_**·**^-^ generation [57]. Quercetin protects mouse cardiomyocytes against inflammation, ameliorates myocardial fibrosis, and reduces the incidence of HF by activating SIRT5, which promotes IDH2 desuccinylation and maintains mitochondrial homeostasis [44]. N-succinylated chitosan and mangiferin (NSC-MGF) nanoconjugates have been used to improve drug targeting by enhancing the bioavailability of antioxidants (e.g., superoxide dismutase and catalase) for the treatment of ischemia-reperfusion injury [85]. 5-Aminolevulinic acid could compensate for the excessive consumption of succinyl-CoA, thereby increasing the overall succinylation level of mitochondrial proteins and improving myocardial energy metabolism [26]. Additionally, active substances derived from traditional Chinese medicine, such as astragaloside IV [89], resveratrol [90], and flavonoids, exert protective effects in other diseases and organisms by modulating succinylation. Collectively, these findings suggest that optimizing drug design and delivery systems targeting the succinylation metabolic axis has synergistic therapeutic potential, warranting further discovery of novel targets and clinical translation.

**Table 2:** Summarizes succinylation-targeted strategies and their clinical trial outcomes.

Drugs	Targets	Disease	Mechanisms	Research and development stage	Ref.
NAD^+^ injection	SIRT5	MI/RI	Promoting SIRT5-mediated SDH-a desuccinylation, decreasing the activity of SDH-a, attenuating the succinate accumulation during ischemia, and finally alleviated ROS production (predominantly O_2_**·**^-^).	Already listed	[57]
Quercetin	SIRT5	HF	Activating SIRT5 to promote IDH2 desuccinylation and maintain mitochondrial homeostasis.	For research experiments only	[44]
NSC-MGF nanoconjugate	N-succinyl chitosan	diabetes mediated hyperlipidemia	NSC-MGF lowered plasma cholesterol to ∼37 % and serum triglyceride to ∼61 %; by comparison, prior studies reported only a 10–40 % reduction in serum triglycerides when MGF was used alone.	For research experiments only	[85]
5-aminolevulinic acid	succinyl-CoA	HF	Compensating for succinyl-CoA overconsumption, increasing overall mitochondrial protein succinylation levels, and improving myocardial energy metabolism.	For research experiments only	[26]

Furthermore, in contexts where energy demand increases, such as during endurance exercise, fasting, or pressure overload, an imbalance between succinylation and desuccinylation may impair cardiac energy production, leading to maladaptive hypertrophy and the accelerated progression of cardiac dysfunction. The underlying mechanism may involve SIRT5-mediated desuccinylation, which activates ECHA to ensure energy supply and maintain cardiovascular homeostasis. In the absence of SIRT5, elevated succinylation of ECHA inhibits its activity, resulting in insufficient cardiac energy supply, and triggering myocardial hypertrophy and HF. These findings suggest that lifestyle interventions may influence cardiovascular health by regulating succinylation [42,91].

In conclusion, regarding the “metabolic switch” of succinylation in CVDs, SIRT5, a desuccinylase, plays a complex role, reflecting the challenges associated with its regulation [87]. In diabetic cardiomyopathy, SIRT5 knockout promotes myocardial hypertrophy and diastolic dysfunction in diabetic mice, whereas cardiomyocyte-specific overexpression of Sirt5 alleviates these changes. This indicates that SIRT5 exerts a significant impact on cardiac function; however, the regulatory direction of its activity remains unclear. In AMI, global SIRT5 KO significantly increases infarct size, whereas liver-specific overexpression of SIRT5 exerts a protective effect by promoting the secretion of FGF21 into the bloodstream, thereby improving myocardial energy metabolism [65]. These findings suggest that SIRT5 exerts distinct effects on CVD across different tissue contexts and physiological conditions, and that precise regulation of its activity to achieve effective CVD treatment remains a challenge.

## 5 Future perspectives

Recent advances in computational biology have driven the development of machine learning models to predict protein PTM sites. Thapa et al. [92] innovatively employed deep learning methods and embedding techniques to identify succinylation sites in proteins based on their primary structure, developing a sequence feature-based prediction model. This model provides a computational tool for the high-throughput screening of modification sites. Zeng Ying et al. [93] applied a computational approach named iSuc-ChiDT, which uses statistical difference table encoding and a chi-square decision table classifier to identify succinylation sites. Zhu et al. [94] proposed RLSuccSite, a novel succinylation site prediction model that uses reinforcement learning with a balanced reward and three-peak physicochemical scoring. It captures residue-level contributions more accurately than traditional feature extraction methods. Additionally, several succinylation site prediction methods, such as MLysPRED [95], SuccSPred2.0 [96], pSuc-FFSEA [97], SSKM_Succ [98], and MDCAN-Lys [99], have emerged. These approaches provide novel tools for predicting protein PTMs, with broad application potential across diverse research fields.

Succinylation is a significant PTM in cardiomyocytes; however, the specific mechanisms underlying the occurrence and development of CVDs have not been fully elucidated. Succinylation may be an important regulatory mechanism in the heart under specific stress conditions. It mobilizes and releases reserves by reducing succinylation in cardiomyocytes, enhancing stress resistance, and maintaining cardiometabolic and cardiomyocyte homeostasis. Additionally, a complex crosstalk network exists between succinylation and other PTMs, such as acetylation, ubiquitination, and phosphorylation [64]. Studies have revealed that succinylation of SUCLG2 enhances its stability by inhibiting ubiquitination, whereas SIRT5-mediated desuccinylation promotes its subsequent acetylation modification [100,101]. Metabolic intermediates act as central hubs that integrate diverse PTMs into coordinated regulatory networks. For instance, shifts in the concentrations of key metabolites (e.g., succinyl-CoA and acetyl-CoA) can concurrently modulate the stoichiometry and localization of distinct PTMs, including succinylation, acetylation, and phosphorylation [42]. This metabolite-dependent interplay underscores the bidirectional relationship between cellular metabolic status and dynamic proteomic remodeling, enabling rapid adaptive responses to pathophysiological cues. Therefore, future studies are needed to deepen the exploration of succinylation's enzymatic mechanisms, its substrate specificity, and its interplay with other modification types.

Emerging research has underscored the role of Ksucc as a “metabolic switch” in the pathophysiological mechanisms underlying CVDs, achieved through precise regulation of enzymes activity, localization, and interactions. Specifically, three core ways this “metabolic switch” operates can be highlighted: First, the dynamic balance of succinylation is governed by succinyltransferases (“writers”: CPT1A, α-KGDHC, KAT2A, KAT3B, HAT1) and desuccinylases (“erasers”: SIRT5, SIRT7, CobB, ScCobB2, HDAC1/2/3) [29]; Second, different pathological microenvironments (e.g., oxidative stress) trigger shifts in the succinylation status of target proteins by regulating the concentration of succinylation donors. This implies that the occurrence of succinylation is directly proportional to succinyl-CoA concentration, which determines the direction of modification [26]; Third, succinylation exerts both beneficial and detrimental effects in different CVD conditions. For instance, during the compensatory stage of HF, myocardial energy demand increases, and SIRT5-mediated desuccinylation activates ECHA—a key enzyme in long-chain fatty acid oxidation—by removing its succinyl groups, thereby enhancing fatty acid β-oxidation efficiency and supplying sufficient ATP to the myocardium. However, in the decompensatory stage, the downregulated expression or inhibited activity of SIRT5 in cardiomyocytes leads to imbalanced succinylation of metabolic enzymes, exacerbating ventricular remodeling and cardiac dysfunction. In diabetic cardiomyopathy, SIRT5 mediates the desuccinylation of CPT2 to promote long-chain fatty acid oxidation and reduce lipotoxicity [32], whereas in the hearts of obese patients, excessive desuccinylation may cause hyperactive fatty acid metabolism, resulting in mitochondrial lipid overload and an ROS burst. These findings provide a theoretical basis for the “context-based succinylation regulation strategy” in CVDs.

Future research in this field can be systematically structured into the following interconnected directions by integrating macro-level strategies with cutting-edge technical approaches: First, focusing on the interaction between succinylation and oxidative stress-related proteins may serve as a key determinant of the progression or alleviation of CVDs. This regulatory network centers on redox balance and exhibits biphasic characteristics. As a critical target for modulating redox balance in CVD interventions, succinylation warrants further in-depth investigation. Second, further investigate the mechanisms of succinylation in CVDs, particularly the close association between HFpEF and succinylation. This includes identifying key target sites and signaling pathways to provide a theoretical basis for developing more effective therapeutic agents. Third, more sensitive and accurate detection methods and technologies have been developed to improve the efficiency of succinylation site identification and to explore the dynamic changes in succinylation modifications in specific proteins across different cell types and pathophysiological conditions. Fourth, enhance the research and development of drugs targeting succinylation regulation, seek highly specific and safe compounds, and promote the translation of basic research findings into clinical applications. Fifth, multicenter, large-scale clinical studies should be conducted to validate the efficacy and safety of succinylation modification as a therapeutic target for CVDs, thereby providing new approaches for clinical management. Specifically, integrating single-cell succinylproteomics with spatial transcriptomics could decipher cell type-specific modification patterns in atherosclerotic plaques, addressing the heterogeneity challenge in tissue-level analyses. CRISPR-mediated site-specific succinylation mimicry enables the functional validation of key lysine residues in myocardial energy metabolism, whereas AI-driven drug design platforms may accelerate the discovery of SIRT5 allosteric activators with tissue-selective efficacy [102]. These technological innovations have bridged the gap between basic modification profiling and translational therapy.

With the rapid development of mass spectrometry, antibody engineering, and gene-editing technologies, research on succinylation has transitioned from phenomenological descriptions to an in-depth exploration of mechanisms and targeted interventions. Future studies must address core challenges, including the functional validation of modification sites, tissue specificity, and dynamic regulatory networks, thereby accelerating the clinical translation of novel therapeutic strategies, such as SIRT5 agonists. Meanwhile, integrating multi-omics analyses with artificial intelligence prediction platforms will expedite the application of succinylation modifications in cardiovascular precision medicine, providing “novel” solutions for this “age-old” family of diseases.

## 6 Conclusion

Ksucc is a fundamental regulatory mechanism that integrates metabolic flux and cardiovascular function, and more crucially, it acts as a “metabolic switch” that modulates redox balance—its mechanisms disrupting redox balance drive the progression of CVDs, whereas those maintaining this balance alleviate disease symptoms. This “switch” function ranges from acute ischemic injury to chronic maladaptive remodeling, positioning succinylation as both a pathogenic mediator and a promising therapeutic target. Notably, the uniqueness of this “metabolic switch” is reflected in the fact that moderate desuccinylation of “protective targets” (e.g., IDH2, ECHA) typically exerts beneficial effects, whereas excessive succinylation or dysregulated modification of “functional targets” (e.g., succinate accumulation associated with SDH) tends to exacerbate pathological progression. This also provides a theoretical basis for the “context-based succinylation regulation strategy in CVDs.” Furthermore, current evidence suggests that SIRT5 is a crucial cardioprotective desuccinylase, whose activity preserves metabolic flexibility and mitigates myocardial damage. As analytical and therapeutic technologies advance, targeting succinylation dynamics will offer innovative approaches for precise cardiology, moving beyond conventional strategies toward metabolism-modifying therapies tailored to individual disease phenotypes. Future efforts should bridge mechanistic insights with clinical validation and ultimately translate this burgeoning knowledge into improved cardiovascular outcomes.

## Funding

This work was supported by the 10.13039/501100001809National Natural Science Foundation of China (No. 82574767,82400418), the health Research and Innovation Capacity EnhancementProgramme of Shaanxi Province (2023 PT-10), Innovation Capacity Support Programme of Shaanxi Province (2023-CX-TD-76), the program of the Clinical Medicine and Pharmacy Research Center, Air Force Medical University, China (LHJJ2023-YX03).

## CRediT authorship contribution statement

**Fei Mu:** Funding acquisition, Investigation, Visualization, Writing – original draft, Writing – review & editing. **Haiyue Zhang:** Investigation, Visualization, Writing – original draft, Writing – review & editing. **Rui Gong:** Conceptualization, Funding acquisition, Writing – original draft, Writing – review & editing. **Rui Lin:** Investigation. **Meina Zhao:** Investigation. **Xingru Tao:** Investigation. **Lei Shang:** Supervision. **Miaomiao Xi:** Writing – review & editing. **Jinyi Zhao:** Supervision, Writing – review & editing. **Jingwen Wang:** Conceptualization, Project administration, Supervision.

## Declaration of competing interest

The authors declare that they have no known competing financial interests or personal relationships that could have appeared to influence the work reported in this paper.

## Data Availability

No data was used for the research described in the article.
